# New Cupping Instrument and Breast Pump

**Published:** 1853-01

**Authors:** S. C. Rogers

**Affiliations:** Addison, N. Y.


### *New Cupping Instrument and Breast Pump.*

Dear Sir:—In making some experiments with a broken syringe, I made the following discovery, which may be of service to the profession, and to the community at large, if it should come to their knowledge. It is this:

A glass tube, one inch in diameter, and six inches in length, with an open mouth of a proper shape, and a piston fitted to the caliber of the tube, thus constituting an air pump, on placing the mouth over the nipple, makes the very best instrument for elongating the nipple, or for empting the breast of its contents that I have ever seen. It works like a charm.

Also tubes of various sizes, with proper mouths, make the best instruments for cupping of which I have any knowledge. The operation of cupping with this instrument is greatly facilitated.

You may make such use of these facts as to you may seem proper.

I have never ordered any of these instruments to be made at the glass manufactories, but have some tubes with which I have made tests that afford much satisfaction to me. Yours, &c.,

Addison, N. Y., Dec. 7, 1852. S. C. ROGERS, M. D.

